# On the Shoulders of Giants—Reaching for Nitrogenase

**DOI:** 10.3390/molecules28247959

**Published:** 2023-12-05

**Authors:** Oliver Einsle

**Affiliations:** Institute of Biochemistry, Albert-Ludwigs-Universität Freiburg, Albertstrasse 21, 79104 Freiburg im Breisgau, Germany; einsle@biochemie.uni-freiburg.de; Tel.: +49-(761)-203-6059

**Keywords:** nitrogen fixation, molybdenum, nitrogenase, enzyme mechanism, vanadium, energy conversion

## Abstract

Only a single enzyme system—nitrogenase—carries out the conversion of atmospheric N_2_ into bioavailable ammonium, an essential prerequisite for all organismic life. The reduction of this inert substrate at ambient conditions poses unique catalytic challenges that strain our mechanistic understanding even after decades of intense research. Structural biology has added its part to this greater tapestry, and in this review, I provide a personal (and highly biased) summary of the parts of the story to which I had the privilege to contribute. It focuses on the crystallographic analysis of the three isoforms of nitrogenases at high resolution and the binding of ligands and inhibitors to the active-site cofactors of the enzyme. In conjunction with the wealth of available biochemical, biophysical, and spectroscopic data on the protein, this has led us to a mechanistic hypothesis based on an elementary mechanism of repetitive hydride formation and insertion.

## 1. Introduction: You Cannot Fix Nitrogen with an Enzyme

The reductive fixation of atmospheric nitrogen by a single known enzyme, nitrogenase, is a story of many heroes, sung and unsung, and a roller-coaster ride of discoveries, disappointments, frustrations, and break-throughs that started as far back as 1888, when Hellriegel and Willfarth reported nitrogen fixation by legumes and graminees [1]. This turned out to be a bacterial process, and in 1903, Jacob Lipman described a free-living soil bacterium he found near Vineland, New Jersey, to be a ‘diazotroph’, a N_2_-eater [2]. This organism, *Azotobacter vinelandii*, remains the most extensively studied model for biological nitrogen fixation to this day, as it is easily cultivated as an obligate aerobe that produces high amounts of nitrogenase during N-limited growth. 

In more than one way, the term ‘biological nitrogen fixation’ is an oxymoron, and nitrogenase, the only known enzyme that catalyzes it, should not be able to do so. Life is a play that is enacted within a rather small window of biophysical boundaries and is based on a few fundamental principles that are indisputable. All organisms are energy-converting machines that take up one form of energy from their environment and convert it into something useful, most prominently the phosphodiester bonds of adenosine-5′-triphosphate (ATP), our basic currency of metabolic energy. In essence, however, we are electrochemical cells that gain energy from the oxidation of nutrients and store it by reducing oxidized compounds. The actual energetic small change that we are shuffling around is electrons that are transferred between redox couples of different midpoint potentials, back and forth, as long as we live, as doing this is the actual definition of being alive. Life at some stage then made the—questionable—decision to leave the oceans, but even so, our cells have remained complex chemical reactors that operate in an aqueous, mildly saline milieu, around neutral pH, and within boundaries of temperature and pressure that allow for water to stay in the liquid phase—‘ambient conditions’, as we usually call it. The protic solvent water, however, puts strict limitations on our energy metabolism. The splitting of water according to

2 H_2_O → 2 H_2_ + O_2_

requires an overpotential of at least 1.23 V (vs. the standard hydrogen electrode, SHE) at pH 0, but only about 0.82 V vs. SHE at a physiological pH of 7. Nowadays, this reaction is widely discussed as a means of storing solar energy as fuel H_2_, but its biochemical relevance is that in an aqueous milieu, cells should not be able to reduce any substrate that requires a more negative potential than 0.82 V, corresponding to just below 80 kJ·mol^–1^ for a single-electron reduction. Electrons with a more negative potential much rather reduce water, which is always present in high excess. Evolution over time has splendidly exploited this ‘water window’, optimally so in the aerobic respiration of our mitochondria, which is the most efficient biological energy metabolism we know. However, there are some essential ingredients to the soup of life that cannot be accessed under these conditions, and the most prominent of these finally gets us back on track: The element nitrogen is a key requirement for building biological macromolecules such as nucleic acids and proteins, but almost all (>99%) of the nitrogen in Earth’s biosphere is present in the form of the chemically inert dinitrogen gas, N_2_, that makes up 78% of our atmosphere [3]. Triple-bonded N_2_ has a bond dissociation energy of −946 kJ mol^−1^, which implies that its reduction requires an overpotential of at least 1.63 V. Reduction of nitrogen at ambient conditions in an aqueous milieu should not be possible, and nitrogenase, which does just that is an impossible enzyme. Enzymes surely have their little tricks and cheats to get things going, but at this magnitude, thermodynamics are not negotiable. The problem is only emphasized by the prominent Haber–Bosch process of industrial nitrogen fixation, invented in 1906 and first established at BASF in 1913 [4], which takes LeChatelier’s principle to the task and reacts N_2_ and H_2_ gas at an iron catalyst surface at temperatures of 400 to 650 °C and 200–400 atm pressure.

N_2_ + 3 H_2_ → 2 NH_3_

This surely works famously well, but it is no option for a microorganism in need of bioavailable ammonium cations.

## 2. Have You Tried to Crystallize the Protein?

Biological nitrogen fixation was one of my smaller worries in 1992 as a student of Biology at the University of Konstanz in the very south of what had just recently ceased to be ‘West’ Germany. I recall a day in a lab course on plant biochemistry run by the team of Peter Böger [5] when there was a buzz of excitement among the graduate students who served as teaching assistants. The experiment of the day was about biological nitrogen fixation, monitoring oxidative stress on the ammonia production by a diazotrophic cyanobacterium that was catalyzed by nitrogenase, and obviously, someone in the United States had produced a crystal structure of this enzyme from *Azotobacter vinelandii* that defied all expectations, revealing am unprecedented catalytic center that must somehow be key to the unique abilities of this system [6,7]. At the time, enzymology and mechanism were on everyone’s agenda in Konstanz, and so I became part of the first generation of students who had to learn to draw this Escher-like two-dimensional projection of the FeMo cofactor of nitrogenase on paper for my exam. 

**Figure 1:**
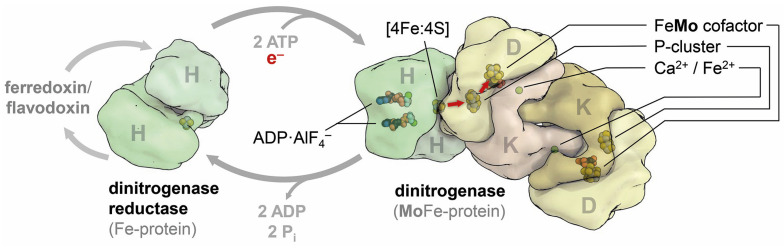
Components of the nitrogenase system and their interaction. A low-potential ferredoxin of flavodoxin reduces the reductase component (or Fe protein), which then binds 2 ATP and undergoes a conformational change that allows for complex formation with the dinitrogenase component. This triggers ATP hydrolysis, upon which an electron is transferred from the [8Fe:7S] P-cluster to the active-site cofactor, a [Mo:7Fe:9S:C]:homocitrate cluster in the case of Mo-nitrogenase (red arrows). The reduction of a single N_2_ molecule to 2 NH_4_^+^ requires this cycle to be repeated eight times at the expense of 16 ATP.

The structure of nitrogenase was ground-breaking indeed, and the one who had achieved this was, of course, Doug Rees at Caltech. In short succession, the Rees group reported the architecture of the nitrogenase clusters [6], the three-dimensional structure of MoFe protein [7], and the one for Fe protein [8], the second component of the nitrogenase system. Crystal structures were a luxury at the time, the product of hard work both in the laboratory and on the computer, and the information they could provide was truly invaluable. Nitrogenase was first isolated in 1966 by Bulen and LeComte [9], and a wealth of physiological and biochemical information has been accumulated since. Structural Biology now brought many of these findings together. The nitrogenase system consists of two component proteins; the dinitrogenase reductase binds and hydrolyzes ATP and provides low-potential electrons by forming a transient complex with the second component, dinitrogenase (Figure 1). Three isoforms of nitrogenases are known, utilizing Mo, V, or Fe as the apical ion to their active-site cofactor, and under conditions of low electron flux and high pN_2_, all catalyze the reduction of dinitrogen according to

N_2_ + 10 H^+^ + 8 e^−^ + 16 ATP → 2 NH_4_^+^ + H_2_ + 16 [ADP + P_i_].

In this reaction, electron transfer occurs sequentially, and the stoichiometric by-product H_2_ is of critical mechanistic relevance, as discussed below. The Rees structures had clarified the architecture of the metal cofactors, finding that both the electron-transferring P-cluster and the catalytic FeMo cofactor are unique to this enzyme, and the general expectation at the time was that this information would advance understanding of biological nitrogen fixation to a similar degree as the structure of the photosynthetic reaction center of *Rhodopseudomonas viridis* had for photosynthesis a few years prior [10].

In my studies in Konstanz, I moved on from this first encounter with nitrogenase, but not too far. During my last year, I worked as a student assistant in the group of Peter Kroneck, together with his graduate student Frank Neese, whose day job was to study the copper enzyme nitrous oxide reductase while teaching himself quantum chemistry in his spare time to move on to become one of the world leaders in this field [11]. During this time, I heard a lecture by Albrecht Messerschmidt from Martinsried, who had collaborated with Peter to investigate ascorbate oxidase, one of the first multicopper oxidases characterized at this level [12,13]. I was fascinated with protein crystallography as a method, and Peter and Albrecht soon offered me the opportunity to perform my diploma thesis work in Martinsried, in the Department of Structural Research headed by Robert Huber, who had been awarded the 1988 Noble Prize in Chemistry for the structure of the aforementioned photosynthetic reaction center, the first membrane protein crystal structure ever reported [10]. This was the year 1996, and crystallography’s triumphant sweep through the molecular life sciences was just picking up speed. A year prior, Juan Fontecilla-Camps and co-workers solved the first structure of Ni,Fe-hydrogenase [14], and Doug Rees had reported the first tungsten-containing enzyme, AOR [15], followed shortly thereafter by the first molybdoenzyme from Robert’s group [16]. My own task that I carried over into my Ph.D. work with Robert and Peter was the pentaheme cytochrome *c* nitrite reductase, an ammonium-producing enzyme from the energy-conserving pathway of dissimilatory nitrite reduction [17]. At this time, metalloproteins were almost exclusively isolated from their native source, chromatography techniques were less automated, synchrotron radiation sources were far weaker and less reliable than they are now, and computers… well, computers. It was the 1990s. Evans and Sutherland vector graphics machines and VAXstations slowly gave way to incredibly powerful SGI workstations that could refine a small structure in only a day or so. Instead of smartphones, the digital time killer of the day was the Tamagotchi (Google it!). There was no world-wide web, and the Internet was a fledgling, text-based labyrinth for only the nerdiest among us. In our Bavarian lab, every submitted compute job would provide ample time to visit a beer garden with good conscience, and a new structure was the product of an entire doctoral project. Metalloproteins offered the possibility to combine structural and biochemical information with advanced spectroscopic methods to provide a comprehensive picture of a complex metabolic machine. So, after obtaining my Ph.D. in 2000, I knew that my next challenge should be again in the field of metalloprotein structural biology. This was when I remembered nitrogenase. Doug Rees was a household name in Martinsried: He once almost joined Robert’s group as a postdoc to analyze crystals of a certain reaction center but then decided to work with Jim Howard on nitrogen fixation. I contacted Doug Rees at Caltech, went to an interview, and gladly accepted the position he offered me.

## 3. A Closer Look

**Figure 2:**
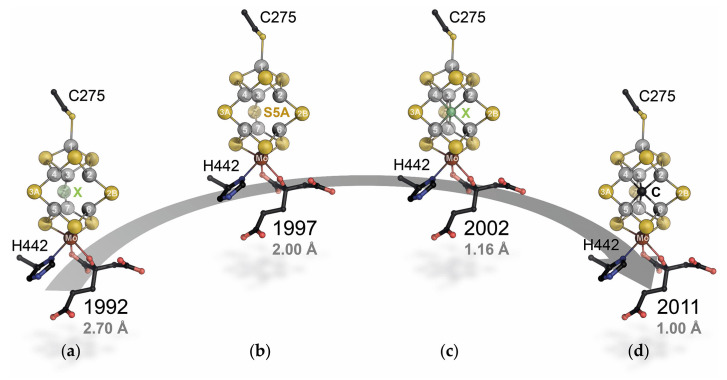
Evolution of structural models for the FeMo cofactor of Mo-nitrogenase. (**a**) The original model by Kim and Rees showed the cofactor with one µ_2_-bridging ligand unassigned [6]. (**b**) Improving the structure to 2.0 Å resolution, the bridging ligand was identified as a third bridging sulfide, S5A [18]. (**c**) Only with an atomic-resolution structure, the Fourier series termination artifacts caused by the high-symmetry structure of the cluster were overcome and a central light atom was revealed [19]. (**d**) Through a combination of HERFD-XAS, ESEEM, and high-resolution crystallography, the central light atom was identified as a carbide in 2011 [21,22].

With an entire laboratory dedicated to the isolation and analysis of nitrogenase, the Rees group had a well-operated setup that allowed me to jump right into my work. Together with Susana Andrade, Benedikt Schmid, and Akif Tezcan, and supported by technician Mika Walton, we isolated Mo-nitrogenase and studied aspects of its assembly, complex formation, and function. Crystals at the time were still grown in sealed capillaries within an anoxic glove box, from which they had to be harvested with gloved hands, cryoprotected, and frozen in liquid nitrogen (pure substrate!). This was tedious but not uncommon at the time, and the oxygen sensitivity of nitrogenase was legendary. Soon, however, I decided to put that particular legend to the test. Having worked with O_2_-sensitive N_2_O reductase in Konstanz and Martinsried, we set up regular sitting drop vapor diffusion plates in a glove box. Over lunch, multiple MoFe protein crystals had formed, and it was unproblematic to optimize these into large single crystals of 1–2 mm in length. These crystals diffracted to almost 1.1 Å resolution at the Stanford synchrotron source, but initially, we hardly expected more than a cosmetic improvement over the then-available 2.0 Å structure of the enzyme [18]. To our surprise, the high-quality electron density map revealed an additional maximum in the center of the cofactor that we soon understood to be a µ_6_-coordinated light atom at the heart of the cluster that was obfuscated at lower resolution by Fourier series termination artifacts created by the highly symmetric cluster itself [19]. This re-defined our picture of the cofactor architecture (Figure 2), but in terms of understanding nitrogenase catalysis, if anything, it made matters worse, as the light atom occupied the only remaining open coordination site to the metal ions of the cluster. Even at atomic resolution, nitrogenase remained reluctant to reveal its inner secrets. Another outstanding feature of the Rees lab was Jim Howard. Having retired from the University of Minnesota, Doug’s former postdoc advisor understandably spent the winter months in Southern California, and during this time, Jim was fully involved in the ongoing work in nitrogenase. As a true enzymologist, he was a constant source of ideas and a (very) rigorous experimenter and running activity assays with Jim was a great experience. Jim passed away in 2022 and is dearly missed.

With the brand-new high-resolution data, Doug Rees sent us to the 2002 GRC on Nitrogen Fixation, which turned out to be the last of its name. It was chaired by Brian Hales, who generously gave me an extra speaker slot on Monday morning. On the opening night on Sunday, I learned much about the restrictions for alcoholic beverages on college campuses and received a stern warning from Barry Smith that I would have difficulties convincing the crowd of my findings. Fortunately, there is little ambiguity in atomic-resolution crystal structures, and the news was taken up very well by the crowd. Barry later wrote a wonderful perspective for our paper [20]. At the meeting, I emphasized the result of our analysis that this central atom is most likely nitrogen more forcefully than we did in the publication, and this, of course, later turned out to be incorrect—the electron density anomaly that hid the central atom at resolutions below 1.5 Å (Figure 2b) would also enhance it slightly at 1.16 Å—but the presence of a central light atom did much to explain the unusual stability of the cofactor that can be extracted in an intact state from the enzyme and was very well received in particular by synthetic and theoretical chemists (Figure 2c). The other part of the experience of this first GRC I attended was to meet many of the literal giants on whose shoulders we stood, starting to make our own contributions. There were David Lowe and Roger Thorneley, Barry Smith and Bob Eady, Bill Newton and Dennis Dean, Dimitri Coucouvanis, Vince Huynh, Lance Seefeldt, Brian Hoffman, Paul Ludden, Stephen Cramer, Dick Cammack, Mike Johnson, Juan Fontecilla, and many more. And, among them, many juniors proved that although the meeting changed its name (to iron–sulfur enzymes and later to metallocofactors), the torch of nitrogenase research was carried on. Patricia dos Santos, Markus Ribbe, Luis Rubio, John Peters, Cathy Drennan, Pat Holland, and Akif Tezcan were early in their careers and have since done so much to explore new avenues of nitrogenase mechanism and application. They are giants standing on the shoulders of giants, which is not necessarily the most stable situation, but it sure is motivating to look around you and make sure that you will not only see knees. The work of the participants of this meeting and of many others has provided an incredible wealth of high-quality data, and part of what makes the current state of the field today so exciting is that, in many cases, we can now reap the harvest of their efforts and connect the dots to obtain a comprehensive picture of what happens during catalysis in nitrogenase.

From Pasadena, I accepted a position as a junior (assistant) professor at the University of Göttingen, Germany, in 2003. The junior professorships were a new career path in the German academic system that no one really had a clear concept of, me least of all. I was, however, in the lucky position to work in the newly established Department of Molecular Structural Biology, headed by Ralf Ficner, who provided all the support and resources that a starting group leader could ask for. With my own lab, our focus shifted away from nitrogenase for the time being as we studied various membrane proteins and transporters involved in the nitrogen cycle. I kept following the nitrogenase field closely but did not contribute. During this time, Tezcan and Rees published their excellent work on the complex formation between different nitrogenase components [23], Ribbe and Hu [24,25,26], as well as Rubio [27,28] studied the assembly of the nitrogenase components, and Seefeldt, Hoffmann and Dean drew the first outlines of their proposal of hydride accumulation on the cofactor that would be a cornerstone of our current mechanistic understanding [29]. Many other important findings were made in this flourishing field during this time, and when I accepted an offer for a full professorship as chair in Biochemistry at the University of Freiburg in 2008, returning to nitrogenase was high up on my agenda.

## 4. Episode IV: A New Beginning

**Figure 3:**
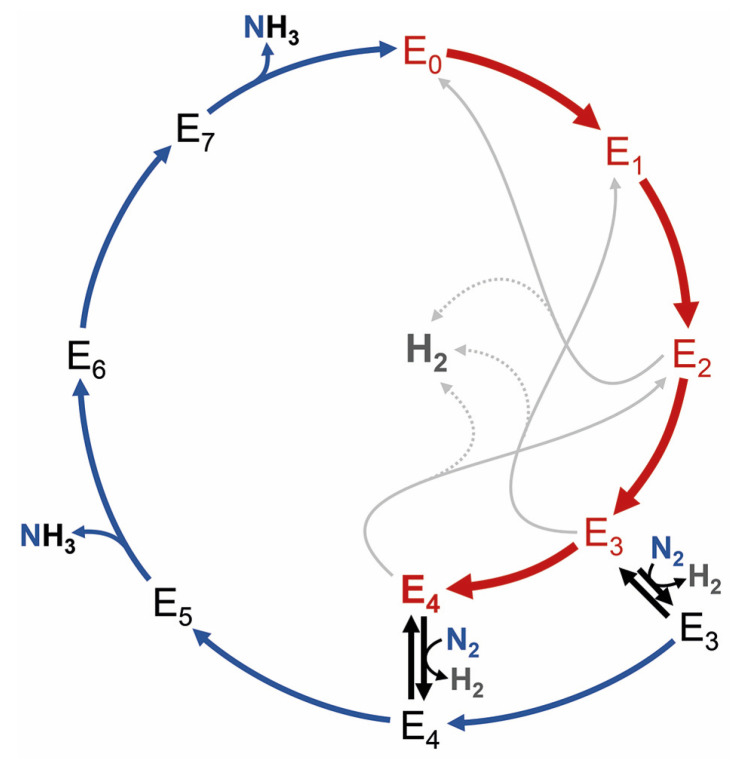
The kinetic scheme for nitrogenase catalysis according to Lowe and Thorneley [37]. The 8-electron process that reduces N_2_ to 2 NH_4_^+^ and releases a stoichiometric H_2_ molecule upon substrate binding is defined in eight states, E_0_–E_7_, that correspond to single-electron transfer events. The enzyme must reach the E_4_ state to bind N_2_ in exchange for H_2_, which is interpreted as a reductive elimination that leaves the enzyme in a super-reduced state.

Working once more in a chemistry department, we built up cell growth, anoxic protein biochemistry, and protein crystallography, and most importantly, I was able to attract many talented, bright, and highly dedicated students to work on the various aspects of structural bioinorganic chemistry that we wanted to address. The first question was about the actual nature of the central light atom I had found in the Rees lab. Brian Hoffman’s group has since provided data to show that our initial interpretation as a nitrogen species was not correct [30,31], and three years down the road, almost a decade after its discovery, Kyle Lancaster and Serena DeBeer at Cornell studied MoFe protein by HERFD-XAS, while we kept working on atomic-resolution X-ray crystallography and ESEEM spectroscopy in collaboration with Stefan Weber. We frequently discussed our data and realized that it all pointed at the central atom being carbon. Teaming up, these results were then condensed into a back-to-back study [21,22]. Shortly thereafter, Markus Ribbe and Yilin Hu, who had provided protein for Serena’s work, showed that this interstitial carbide originated from S-adenosyl methionine and was inserted by the radical/SAM enzyme NifB, another unique reaction in the nitrogenase world [32]. As exciting as these findings were for the experimentalists, they underlined once more that most work so far had only addressed the resting state of Mo-nitrogenase, where there is no free coordination site on any of the metal ions. The mode and position of nitrogen binding were still up for grabs, and the mechanism of biological nitrogen fixation remained as enigmatic as ever. Twenty years after the first crystal structure of Mo-nitrogenase, the gold standard framework for nitrogenase catalysis still was the epochal enzymological analysis by David Lowe and Roger Thorneley that led to a kinetic scheme for the enzyme with eight intermediate states representing the individual electron transfer steps [33,34,35,36] (Figure 3). Structural biology had contributed much to our understanding of the resting state, E_0_, but little beyond. 

The new and complete structure of the cofactor also triggered multiple theory groups to study its electronic structure and properties, but the difficulties in treating such complex systems led to quite divergent mechanistic proposals. The road forward was not immediately apparent. In its resting state, nitrogenase does not bind substrates, and according to Lowe and Thorneley (Figure 3), it will not even bind N_2_ prior to reaching E_4_, a high-energy state that will be extremely hard to access by structural analysis. We, therefore, proceeded to further characterize the electronic structure of the FeMo cofactor to provide a better basis for theory and to help us better understand what makes this metal site so unique. It was time to pick up on some unfinished business from the Caltech days. The first of these was an EPR study using single crystals of MoFe protein, aiming to derive a spatial correlation between the pseudo-*D*_3_ symmetric structure of the FeMo cofactor and the apparent *S* = 3/2 *g*-tensor of its resting state E_0_. Susana Andrade and Thomas Spatzal collected EPR and diffraction data for Mo-nitrogenase, showing that the rhombic *g*-tensor indeed aligned specifically such that its longest axis was collinear with the pseudo-three-fold of the cofactor. We noted that the other two principal axes of the apparent *g*-tensor that were very different with *g*_y_ = 3.65 and *g*_x_ = 2.01 were oriented to highlight two irons of the cofactor, Fe2 and Fe6, in the direction of *g*_x_, while *g*_y_ was in a plane formed by four others, Fe3, Fe4, Fe5, and Fe7 [38]. Like many findings on nitrogenase, this first indication of a special role for Fe2 and Fe6 would take years more to gain significance. With Doug Rees, I expanded on an idea that Holger Dobbek had conceived during our time as graduate students in Martinsried: As the anomalous scattering contribution Δ*f*″ of an atom of a given element is proportional to its absorption cross-section for X-rays, it should change among diffraction datasets collected along an X-ray absorption edge for this element. With the added spatial resolution of a diffraction experiment, this allows for the reconstruction of an individual X-ray absorption curve for each scattering atom of a given type. While such data are not as highly resolved as X-ray absorption spectroscopy, our proof-of-principle study on a [2Fe:2Fe] ferredoxin showed that differences such as oxidation states can be very well distinguished [39]. In application to nitrogenase, Thomas Spatzal collected a series of diffraction datasets along the K-edge of iron and refined the individual Δ*f*″ contributions for each iron site at every wavelength [40]. At the same time, Ragnar Björnsson and Serena DeBeer, now a director at the Max Planck Institute for Chemical Energy Conversion, reinvestigated the single molybdenum ion in the cluster by Mo-K-edge XAS. Her data provided strong support for a Mo(III) ion in the cluster, more reduced than any other known Mo site in biology, where the metal typically oscillates between Mo(IV) and Mo(VI) [41]. Our spatially resolved anomalous dispersion analysis (SpReAD) complemented this finding by assigning three of the seven iron ions (Fe1, Fe3, and Fe7) as more reduced than the other four (Fe2, Fe4, Fe5, and Fe6). Assuming a formal oxidation state of Fe(II) for the more reduced sites and Fe(III) for the oxidized positions, this could be integrated with data from 2002 by Noodleman and co-workers, who used broken-symmetry DFT to find that an arrangement of the high-spin metal centers that maximizes antiferromagnetic coupling was the most stable [42]. Their top broken-symmetry solution, BS7, combined with Serena DeBeer’s Mo(III) and our distribution of Fe oxidation states yielded a total spin state of *S* = 3/2, in line with spectroscopic data (vide infra). I did like this result so much that it almost made me forget that once more, we did not gain much insight into nitrogenase function, staring anxiously at a resting state that still refused to reveal any details of its interaction with N_2_.

## 5. Family Business: The Three Nitrogenase Isoforms

**Figure 4:**
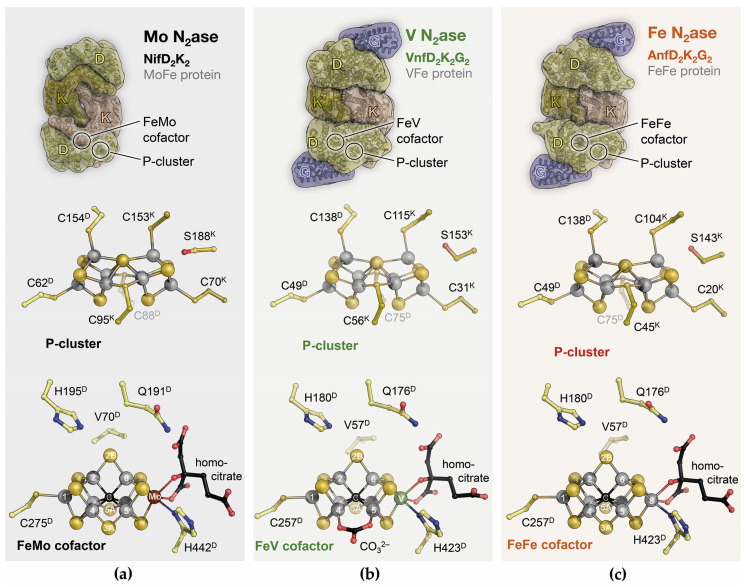
The three variants of nitrogenase. (**a**) Mo-nitrogenase is encoded by *nif* genes and consists of the Fe protein NifH_2_ and the MoFe protein NifD_2_K_2_ (above). MoFe protein contains [8Fe:7S] P-clusters at the DK interfaces (middle) and an active-site FeMo cofactor (below). This cofactor is a pseudo-*D*_32_ symmetric [Mo:7Fe:9S:C]:homocitrate cluster attached to the protein only through its apical metal ions. (**b**) In the alternative V-nitrogenase, encoded by *vnf* genes, the Fe protein VnfH_2_ works in conjunction with the VFe protein VnfD_2_K_2_G_2_ (above). The P-cluster corresponds fully to that of the MoFe protein (middle), and the active-site FeV cofactor had Mo replaced for V, as expected (below). What was unexpected was the replacement of one µ_2_-sulfide, S3A, by a carbonate anion that is not exchanged during catalysis. (**c**) In Fe-nitrogenase, *anf* genes encode the Fe protein AnfH_2_ and the FeFe protein Anf D_2_K_2_G_2_ (above). Although FeFe and VFe proteins are closely related, their Fe proteins AnfH and VnfH are more distinct than VnfH and NifH. The P-cluster of FeFe protein is highly similar to those of the isoenzymes (middle), and the FeFe cofactor (below) is a truly *D*_32_ symmetric [8Fe:9S:C]:homocitrate cluster that likely corresponds to a precursor (L-cluster) of the other sites.

Nitrogenase was initially characterized as a molybdenum-containing enzyme [43], but it soon became apparent that it differed from all other known Mo-dependent enzymes in that it did not contain an organic molybdopterin cofactor but had a molybdenum ion as part of an iron–sulfur-based metal cluster (Figure 4a). Only in 1980 did Paul Bishop present evidence for an alternative nitrogen fixation system that was dependent on vanadium [44], and with Bob Eady, he showed that this system had its own structural genes and isolated the enzyme in 1986 [45], as did Hales and co-workers [46]. In 1988, Bishop discovered that a third variant of the nitrogenase system could be isolated from a Δ*nifHDK* strain, whose activity did not depend on any metal other than iron [47]. As finally confirmed by its genome sequence [48], the model diazotroph *A. vinelandii* thus contained three different isoforms of nitrogenase that are closely related in structure and function but show variations that allow for important conclusions regarding the overall mechanism. All three nitrogenase isoforms consist of two component proteins. The Fe proteins NifH, VnfH, and AnfH are the dinitrogenase reductases that bind and hydrolyze 2 ATP for each electron transferred via a [4Fe:4S] cluster situated at the interface of the 60 kDa homodimer. The dinitrogenases or MFe proteins (with M = Mo, V, Fe) are built around a heterotetrameric D_2_K_2_ core that, in the case of VFe protein and FeFe protein, is extended by two copies of a G subunit. All dinitrogenases contain an electron-transferring [8Fe:7S] P-cluster at the interface of the structurally related D and K subunits, and an active-site cofactor buried deeply within the D subunits (Figure 4). This cofactor is the enigmatic site of N_2_ reduction, and while the general expectation was that in the isoenzymes, the apical Mo ion of FeMo cofactor was replaced by V or Fe, respectively, any changes in the cofactor environment that might account for the observed differences in substrate specificity and reactivity were unknown. The two alternative nitrogenases are considerably less stable than Mo-nitrogenase and are only produced in the absence of the respective heterometals: Mo repressed V- and Fe-nitrogenase, and V repressed the Fe-dependent enzyme. Our initial attempts at crystallizing a tagged VFe protein from a Δ*nifHDK* strain of *A. vinelandii* in collaboration with Markus Ribbe and Yilin Hu were unsuccessful, so we decided to optimize the growth conditions for native *A. vinelandii* to maximize the production of V-nitrogenase. This process and the subsequent crystallization of the enzyme proved highly challenging [49], and it took more than four years of diligent laboratory work and perseverance by Daniel Sippel to solve the structure of VFe protein and be rewarded with true atomic resolution and unexpected insights into the structural details of the enzyme (Figure 4b). First, we noted that the expected replacement of Mo by V was not the only change at the FeV cofactor. In addition, one of the µ_2_-bridging sulfide ions at the cluster belt was replaced by a divalent carbonate anion, whose origin and specific role remain unknown [50]. A second finding in VFe protein was far more consequential for understanding nitrogenase catalysis: In addition to the constitutive replacement of one sulfide by carbonate, another µ_2_-sulfide, termed S2B and bridging Fe2 and Fe6 of the cofactor, was labile and replaced to varying degrees by a single light atom [51]. This binding corresponded to the one observed for the inhibitor CO (vide infra), but the smaller, monoatomic ligand now allowed for the side chain of a conserved glutamine residue near the active site to flip, opening on one side a holding position for the released sulfide S2B and forming a short hydrogen bond to a similarly conserved histidine residue right above the Fe2-Fe6 edge of the cluster. Interestingly, the histidine residue is suggested to serve as a proton source during N_2_ reduction and is connected to the protein surface via a tight network of hydrogen bonds. For the subsequent integration of a mechanism, the blocking of this proton source when a small ligand binds to the cofactor is essential.

The two key lessons from this structure were that both the active-site cofactor and its surroundings are structurally flexible and that sulfide S2B can be reversibly replaced by a bridging light atom. Following a tradition, we assigned this light atom as a nitrogen species, NH, implying that it might be an intermediate of N_2_ reduction [51]. Shortly thereafter, this was contested based on a re-analysis of our electron density map [52] and DFT calculations [53] that both favored OH, a bridging hydroxyl. In this case, however, the debate is misleading. The key aspect of replacing sulfide S2B is that it creates a coordination site for substrates and—as we will see—inhibitors that were crucially absent in the resting state of the cofactor. The light atom may be a nitrogen species, it may be oxygen (although this should not originate from O_2_), or it may even be a carbon species, as it should constitute an intermediate of the respective reduction reaction catalyzed by the enzyme. We do not yet understand why this conformation was seen in V-nitrogenase but so far not in a Mo-nitrogenase, but further support for the key role of this position in catalysis recently came from our structural analysis of the third isoform, Fe-nitrogenase. Extending on our strategy of metal depletion developed to produce V-nitrogenase, we worked with Mo- and V-depleted cultures of *A. vinelandii* to induce the expression of the *anf* genes encoding for this enzyme. The three nitrogenases decrease in their activity Mo > V > Fe, and this also correlates to a reduction in both their stability and sensitivity to O_2_. It was no small feat, and another 4 ½ years of work, that Christian Trncik succeeded in isolating, characterizing, and crystallizing both the Fe protein AnfH [54] and the nitrogenase FeFe protein (Figure 4c) [55]. Fe- and V-nitrogenases of *A. vinelandii* are more closely related to each other than to the Mo-dependent enzyme, and while the active site held the expected [8Fe:9S:C]:homocitrate FeFe cofactor, it showed a dual conformation of a resting and a turnover state with partial replacement of sulfide S2B that we had also observed in VFe protein [56]. The determination of a three-dimensional structure is always only one aspect of understanding the intricacies of a macromolecular machine, but when solving the structures of the two alternative nitrogenases, I was reminded of the appeal of having this singular moment of discovery when looking at a new electron density map for the first time. All the new information in a structure is revealed at the push of a button, and one can spend time rummaging through its complexity for many hours, discovering fundamental principles and unknown details, always ready to stumble across a surprise around the next turn.

## 6. Alternative Substrates: Learning from CO about N_2_

**Figure 5:**
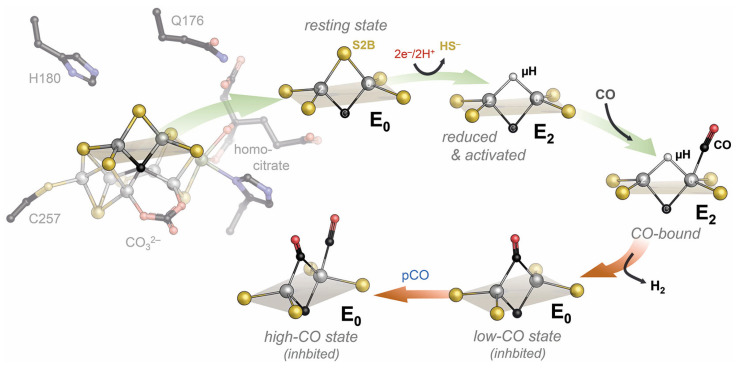
CO inhibition of the nitrogenase cofactor. Using the FeV cofactor as an example, the resting state is reduced by two electrons, leading to the release of sulfide S2B and its replacement by a bridging hydride in the E_2_ state. Here, CO can bind terminally to the t-site at Fe6. If the µ-hydride is accidentally protonated and lost as H_2_, the CO ligand can migrate to the µ-site, but the enzyme is formally returned to the E_0_ state, now in a CO-inhibited form. Pressurization of crystals of this low-CO state with CO gas led to the structure of the high-CO state that still is an off-pathway resting state E_0_, but with two CO ligands that highlight the µ- and t-site for substrate/intermediate binding.

The reduction of N_2_ is the most challenging and unique catalytic ability of the nitrogenase enzymes. The Lowe–Thorneley scheme dictates that four electrons must be accumulated on the cofactor, and the accumulation of surface hydrides suggested by Seefeldt, Hoffman, and Dean would then allow for the reductive elimination of H_2_ that leaves the cofactor in a super-reduced state representing the catalytically active species [57,58,59]. The difficulty of even reaching this critical E_4_ state is highlighted by the high rate of H_2_ production by nitrogenase—at least in vitro—that represents an abortive side reaction when a surface hydride is accidentally protonated, and two of the accumulated electrons are lost (Figure 3) [60]. All intermediate states on the way from the resting state to E_4_ are unstable, and the super-reduced E_4_* state after H_2_ elimination clearly is highly elusive, although its fingerprints can be seen, for instance, in deuterium exchange experiments [61,62,63]. Turning a kinetic scheme into a mechanism requires adding precise structural information to each intermediate state, and for nitrogenase, this has been a critical point for a long time. States are short-lived, and hydrogen atoms and hydrides cannot easily be depicted by crystallography. The available theoretical models diverge about the electronic properties of the cofactor and, with that, the sites of substrate/intermediate binding. Alternative substrates of the enzyme, however, are less stable than the N_2_ molecule and may thus be easier to investigate. The most prominent of these alternatives is acetylene, C_2_H_2_, a triple-bonded gas isoelectronic to N_2_ that is reduced to ethylene, C_2_H_4_, in a 2-electron reduction reaction that is used as a common assay using a gas chromatograph [64]. Acetylene reacts with nitrogenase already in the E_2_ state that should be far more accessible than E_4_, but the properties of this reaction are not ideal: Acetylene is not reduced completely to methane, which would be analogous to N_2_ reduction, and the carbon atoms are and remain protonated so that the molecule typically interacts with metals side-on via its nucleophilic triple bond [65]. A closer analog to N_2_ is CO, also with the same number of electrons and high stability, but characterized early on as a non-competitive inhibitor for all known nitrogenase substrates other than protons [66]. This means that its binding diverts all electron flow toward ‘H_2_ production’, i.e., the protonation of hydrides. ‘Non-competitive’ implies inhibitor binding to the enzyme independent of the substrate. It is mostly found in allosteric mechanisms but also applies if the actual substrate binding site is in a different state for inhibitor binding than it must be to interact with substrates: E_2_ for CO vs. E_4_ for N_2_. As an ideal σ-donor and π-acceptor, the strong-field ligand CO binds strongly to many metal sites so that its inhibitory effect on nitrogenase was far less surprising than the finding by Markus Ribbe and Yilin Hu, who reported in 2010 that the alternative, vanadium-containing nitrogenase system reduces CO [67]. Most interestingly, they found that the dominant (>93%) product of the reaction is not fully reduced methane, CH_4_, but rather ethylene, as in the case of acetylene reduction [68]. Thus, the enzyme binds CO and is inhibited by it, but it also has a pathway of activating and reducing the molecule, which involves a C-C bond formation and the release of an unsaturated product. Coincidentally, the product range of this reaction is strongly reminiscent of the industrial Fischer–Tropsch process, which, in terms of catalyst and reaction conditions, is analogous to Haber–Bosch nitrogen fixation. In both cases, it is a particular lattice plane of the crystalline iron catalyst that features the exact interatomic spacings to drive the reaction to the observed outcome [69]. In the enzyme, the observation of C-C coupling from two CO molecules implies two distinct binding sites, which was corroborated by decades of spectroscopic studies that showed states designated ‘low-CO’ and ‘high-CO’, with one molecule of CO binding to the enzyme in the former and at least two in the latter [65,70,71,72,73,74,75,76]. In 2014, my former graduate student Thomas Spatzal, now a postdoc with Doug Rees, then succeeded to inhibit Mo-nitrogenase with CO under turnover conditions, isolate MoFe protein from the mixture, grow crystals, and collect diffraction data to 1.5 Å resolution [77]. I vividly remember that when Doug Rees sent me this structure, I was teaching at the 2014 Penn State Workshop on Bioinorganic Chemistry, and I was immediately struck with what I saw because it just looked so … right. This was the very first observation of a ligand bound to a nitrogenase cofactor, and it was nothing like the distorted clusters or surface associations that were predicted, anticipated, or feared. Instead, the CO molecule had ejected the bridging sulfide S2B that later gained prominence in V- and Fe-nitrogenase. CO replaced this atom, forming a bridging carbonyl at Fe2 and Fe6, a classic in metalloorganic chemistry (Figure 5). Michael Rohde and Katharina Parison (née Grunau) in my group did the same for V-nitrogenase and found the exact same binding mode, which was not obvious considering that only V-nitrogenase can reduce this gas [78]. Having a suitable picture of the low-CO state, attempts at describing the high-CO state were made by pressurizing crystals of the CO-complex with more CO, and again, these were successful for MoFe and VFe protein and were reported in parallel by the Rees group and by us [79,80]. In both cases, the binding of a second molecule of CO was observed, this time as a terminal ligand to Fe6, directly adjacent to the first bridging CO molecule of the low-CO state. The two carbon atoms were at a close distance, and the story of C-C bond formation from this geometry literally wrote itself (Figure 5).

**Figure 6:**
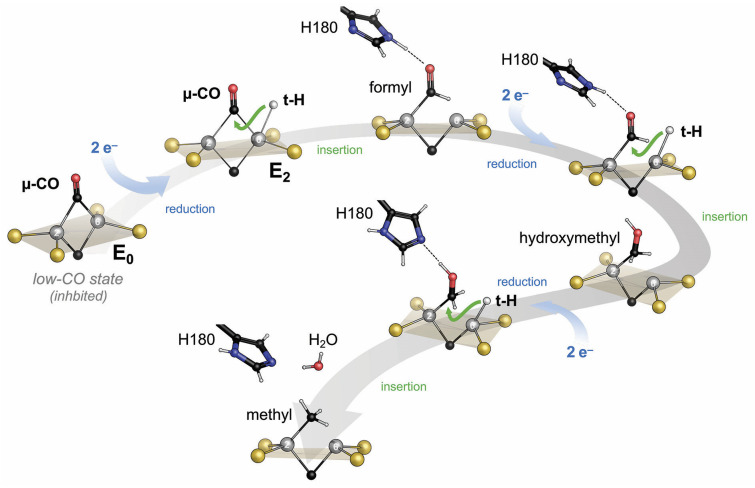
Proposed mechanistic framework for nitrogenase on the example of CO reduction to the methyl stage by V- and Fe-nitrogenase. Two-electron reduction from the resting E_0_ state leads to the formation of a terminal hydride at the t-site, Fe6. This t-H inserts into the bound ligand, and the cycle is repeated, leading through formyl and hydroxymethyl adducts to C-O bond cleavage, water release, and a methyl group bound to the enzyme.

It did so, of course, with a few caveats. One was to clarify what states these CO adducts represented. CO does not bind to the resting state of nitrogenase but only requires the enzyme to reach E_2_ for binding and not E_4,_ as is required for N_2_ reduction. But what is the actual inhibited state? Discussing this question in our group, many of the countless, isolated data points that were amassed on nitrogenase over decades started to fall into place. This story is and remains a hypothesis but integrates the vast majority of what we know [80]. It goes as follows: The nitrogenase cofactors can only be reduced by a single electron. The second, in E_2_, already forms the first hydride on the cluster surface. From our structural data, we have proposed that this hydride forms a bridge between Fe2 and Fe6, essentially replacing sulfide S2B (Figure 5) [81]. Theory agrees that S2B is protonated in E_1,_ and one of its bonds to iron is substantially weakened or broken [82], but the calculations so far do not show a complete dissociation of the resulting dangling thiol. It is this hydride-bound E_2_ state that CO can access, and with the µ-bridging position (that we designate the ‘µ-site’) occupied, the first encounter of CO with the enzyme will be through terminal binding to Fe6 (the ‘t-site’). This complex, then, is where the role of CO is decided. CO can remain bound to the t-site without interacting with the µ-hydride. This will keep the catalytic cycle from progressing forward, and as a result the hydride will eventually be subject to protonation and will be lost as H_2_. The CO ligand in the t-site then is in the ideal position to migrate to the µ-site and form the bridging carbonyl intermediate seen in the crystal structures [77,78]. Importantly, however, this occurs after release of H_2_ from the E_2_ state of the enzyme. The two electrons accumulated to this point are lost, and the enzyme is formally in the resting state E_0_, but with a bound CO ligand (Figure 5). The low-CO state therefore is an inhibited resting state. It is off-pathway for substrate reduction and requires turnover conditions to return to a catalytically competent state [80,81]. Why then is H_2_ production not inhibited by CO? We think that along the same lines, the first interaction of any substrate is to form a terminal ligand at Fe6, the t-site. This is also true if the substrate is a proton and the formed intermediate is a terminal hydride [81]. Terminal hydrides are less stable than bridging ones, and this t-hydride would quickly migrate into the µ-site to form the E_2_ state as described above. In the inhibited state, however, this is prevented by the CO ligand so that the t-hydride is locked in place and will eventually be protonated to form H_2_. Protons are the only substrate of the enzyme that forms a reactive intermediate at the t-site and can, therefore, still be reduced even if the µ-site is occupied. All other substrates must migrate into the µ-site after initially binding to the t-site for reduction to occur. The µ-site is occupied by a hydride so that the substrate *inserts* into the bound hydride, resulting in a concerted two-electron reduction. If this substrate is a t-CO, this is what makes the difference between CO inhibition (loss of the hydride as H_2_) and CO reduction (insertion of CO into the bound µ-hydride, Figure 6). In our proposal, we suggested that from here, two further electron transfers from Fe protein generate another t-hydride at Fe6 that then, in turn, inserts into the bound intermediate so that all reductive steps are two-electron transfers [80]. We cannot exclude that for some or all substrates, it is energetically favorable to directly reduce the bound intermediates rather than form t-hydrides, but this does not affect the essence of the mechanism. The action of nitrogenase thus comes down to a strikingly simple, repetitive two-electron transfer mechanism: A t-hydride is formed and inserted into the µ-site. After four electrons are transferred, the C-O bond is cleaved, water is released, and a methyl group remains bound to the cofactor (Figure 6) [81]. In Fe-nitrogenase, which also reduces CO, this ligand is predominantly protonated and released as methane, while in V-nitrogenase, it stays bound. The reasons for this difference are unclear. In the presence of CO, the VFe protein methyl adduct can then bind another t-CO, which will be inserted into the methyl group, resulting in the enigmatic C-C coupling step. The following two reduction cycles then take this intermediate to bound ethane, which VFe protein again is reluctant to release so that a small amount of longer chain hydrocarbons is formed. For the ethane intermediate, however, the presence of a β-carbon now opens the possibility of a reductive β-hydride elimination to release the product ethylene, leaving a µ-hydride at the cofactor, corresponding to the E_2_ state [80,81].

The proposed action of the enzyme is straightforward, and a terminal hydride as the active reducing species also has the high reducing power (low Δ*E*) that makes this enzyme unique. Nevertheless, nitrogenase is the enzyme of biological nitrogen fixation, so how does this apply to N_2_ reduction that does not require the binding of two molecules of N_2_ to the enzyme and where there is no N-N bond formation (quite the contrary)? How do these processes differ, and is the discussion above at all relevant? The known part is that the E_2_ state I described above is not sufficient to activate the inert N_2_ molecule. The elementary steps of nitrogenase catalysis imply that as the enzyme progresses to E_4_, a second hydride is formed at the t-site [60]. As this second hydride migrates to the µ-site, it might trigger the elimination of H_2_ immediately, leaving the cofactor in a two-electron reduced state that now is sufficiently reducing to break the N_2_ triple bond [51]. However, we disfavor this direct elimination of H_2_, as the resulting intermediate would be highly reactive and too unstable to persist until substrate N_2_ diffuses to the cluster and binds. Instead, taking inspiration from model chemistry by Pat Holland [83], Jonas Peters [84], and others, we suggest that the second hydride forms a *bis*-µ_2_-hydride diamond core with Fe2 and Fe6, possibly triggered by the binding of a N_2_ molecule to the t-site. The two adjacent hydrides then eliminate H_2_, with N_2_ in a perfect position for direct reduction as it migrates into the µ-site [55,60]. This one point where the reductive elimination of H_2_ is used as a catalytic trick to generate a 2-electron-reduced cofactor is the only part of the reaction that is unique to N_2_ fixation. With the triple bond broken, the resulting intermediates can be reduced in full analogy to the steps for CO reduction outlined above, and product NH_4_^+^ is eventually released.

These steps also suggest structures of intermediates, some of which have already been defined by spectroscopy [82,85,86]. It will be very challenging to visualize these intermediates even at the high resolution of current crystal structures, but I emphasize that this mechanism for nitrogenase is not merely the fever dream of a desperate structural biologist. It gains its value from integrating very well with almost all existing data and rationalizes mechanistic peculiarities that have, in some cases, been known for decades. Of course, it also leaves us with open questions and thus highlights the specific points where further study is needed.

## 7. So What about Molybdenum?

Ralf Mendel deserves high praise for putting together this series of essays centered on molybdenum in the living world, and I realize that while the Mo ion in nitrogenase is unique in biology [87], little has been said about its value in the enzyme, other than that some things actually work better without it. Nevertheless, although its biogenesis is the most intricate of the three isoenzymes, Mo-nitrogenase presumably is the evolutionarily oldest one, and it also is the one that all diazotrophs possess. Biological nitrogen fixation dates back far in evolution [88,89] and is found in diazotrophs of the kingdoms of bacteria and archaea but not in eukaryotes. The switch to a V-dependent enzyme may have been triggered by the transition of organisms from marine habitats, where molybdate is quite abundant, to terrestrial ones, where Mo availability is lower, while vanadate presents an alternative. Another hypothesis is that the differences in catalytic abilities, and foremost the reactivity toward CO, may be of physiological relevance so that the alternative enzymes are not (or not only) intended for nitrogen fixation [90]. For a long time, and once in a while still today, the Mo ion of FeMo cofactor was also suspected to be the actual binding site for substrates [91,92], and the possibly labile, organic homocitrate ligand at this site could dissociate to open up a binding site. Current data favor iron, as discussed above, but with nitrogenase, one should always be ready to be surprised. 

**Figure 7:**
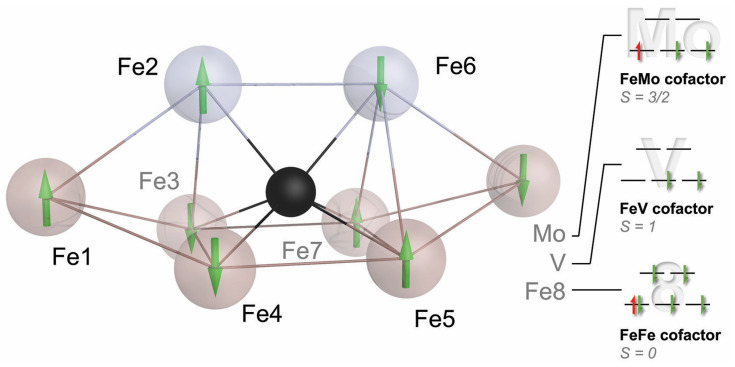
Proposed electronic structure of the resting state E_0_ for the nitrogenase cofactors. Only metals are shown. Sulfides and the central carbide are omitted for clarity. The arrow in each metal indicates the spin orientation for the high-spin systems, following the BS7 coupling scheme. As a common principle, Fe2 and Fe6 emerge as the most oxidized sites, and Fe6 takes up the electron from the Fe protein via the P-cluster. In all clusters, three additional electrons are distributed across Fe1, Fe3, Fe4, Fe5, and Fe7, with delocalization between Fe3–4 and Fe5–7. Only the heterometals or Fe8 in the FeFe cofactor have an octahedral ligand field. In this proposal, the resting state configurations add up to the apparent spins of *S* = 3/2 for FeMo cofactor and integer spins for FeV and FeFe cofactors.

The role of molybdenum thus remains enigmatic, but a closer look at the current model for the electronic structure of the cofactors may provide some hints (Figure 7). The coupling of the high-spin metal centers in the FeMo cofactor is best described by the BS7 coupling scheme [93]. For Fe2 and Fe6, the iron ions involved in substrate binding, this scheme likely establishes antiferromagnetic coupling to all surrounding metals—with one exception. Fe2 couples ferromagnetically with the apical Fe1, and Fe6 does so with the other apex of the cofactor that in Mo- and V-nitrogenase is occupied by the heterometal. In the preceding discussion, I have presented Fe6 as the key player for substrate binding to nitrogenase—a postulate that Dos Santos and colleagues first made based on EPR data from a variant MoFe protein—and this is the exact site that couples most strongly to the heterometal [94]. The electronic structures at this point are more complicated, but even if Mo (or V) are not the metals that bind dinitrogen, their influence on the reactivity of the overall process is immediate and important. The surrounding of the cofactors is strikingly similar in all three isoforms, and the residues in the cluster cavity are fully conserved. It is reasonable and more than likely that many of the observed differences are directly due to the influence of the heterometal. Another interesting implication of this model is that the large multi-metal cofactors only use two iron ions for substrate and hydride formation. What then explains the size of the cofactor and the investment into its biogenesis? Why are there no simpler dinuclear metal sites able to catalyze N_2_ fixation? Looking once more at model complexes, we can hypothesize that an E_4_ state with two bridging hydrides as in the Holland and Peters models [83,84] may even be too stable to efficiently eliminate E_2_ if allowed to relax its metal–metal distance. In the nitrogenase cofactors, the unique Fe_6_:µ_6_-C core with the other µ_2_ bridging ligands in place will restrain the Fe2–Fe6 distance, destabilizing the E_4_ state to favor efficient H_2_ elimination. I have described this carbon-doted iron core of the cofactor as its ‘heart of steel’ to emphasize the relevance of its rigidity, which has been favorably taken up by the community [95].

## 8. What Is Next in Nitrogenase Research?

Necessarily, this review provided a limited perspective on a large, highly complex, and long-worked field of research. I have focused on the level of understanding that we have gained by studying the structures, properties, and interactions of the three known isoforms of nitrogenase, Mo-, V-, and Fe-dependent enzyme systems, seen through the focus of the contributions of my team. The mechanistic hypotheses outlined here are an attempt at integrating as much of the available data as possible, but they do leave some open questions of their own. We do not yet have a suitable understanding of the differences in the reactivity of the isoenzymes. Many of the postulated intermediates could be drawn terminal to Fe2 (as we did) or bridging both irons and the older hypothesis that the first surface hydride forms in the E_2_ state was recently challenged for FeFe protein, where a hydride was detected in E_1_, implying an oxidation of the cofactor [96]. All these points will be addressed, and the field is eagerly awaiting the theory to mature into a reliable arbiter with predictive power for complex metal clusters. An enzyme such as nitrogenase, of course, deserves attention along many other lines of research: The evolutionary history of nitrogen fixation is enigmatic and fascinating, with its origin tracing back to—of all things—enzymes from ancient tetrapyrrole biogenesis pathway, including those for bacteriochlorophyll [97] and for coenzyme F_430_, the unique nickel porphyrin cofactor of methanogenic archaea [98,99]. The relationship and evolution of the extant nitrogenases have inspired attempts at reconstructing ancient nitrogenases through reverse phylogenetic engineering [100], and the Fe proteins as ATP-driven low-potential reductases have been found in a variety of other contexts, including challenging radical reactions [101]. The interplay of Fe proteins and dinitrogenases also goes far beyond a simple electron transfer reaction, and the mechanisms that convert the chemical energy of ATP phosphodiester bond hydrolysis into a lowered midpoint potential of the electron that reaches the active-site cofactor are a busy field of study [102,103]. Another vast area of nitrogenase research is the biogenesis of the enzymes and their metal clusters. The assembly of MoFe protein requires approximately 20 gene products [104], although production of the enzyme from a minimal gene cluster has been reported [105], and even the less intricate Fe-nitrogenase is dependent on a minimal set of nine genes when choosing a suitable expression host [106]. For the dinitrogenase, the P-clusters are inserted into the apoprotein as a pair of [4Fe:4S] clusters and then fused through the action of the Fe protein [25], while the cofactor is assembled ex situ through several states and only then inserted into the enzyme [107]. It is due to this complexity that, to this day, there is no convenient heterologous production system for nitrogenase, although an excellent genetic system for homologous protein engineering in *A. vinelandii* has been established by Dennis Dean and co-workers and is widely in use today [108,109].

Many questions thus remain to be answered, but although this challenge has been compared to the first ascent to a mountain summit [110], the motivation here is not just that ‘it’s there’. Fixing atmospheric nitrogen is a metabolic ability that emerged early in evolution but has never made it into the eukaryotic world. Nitrogen availability quickly becomes a growth-limiting factor if biomass is removed from an environment, i.e., in any modern agricultural setting. This was where the Haber–Bosch process was a game-changer [111] that led to a present where half of the human population can only be sustained through the use of nitrogen fertilizers [112]. Fertilizer use leads to nitrogen pollution, whose mitigation poses severe challenges [113], and an obvious solution is to transfer the ability to use atmospheric N_2_ as a nitrogen source for growth into staple crops. Major efforts are spent to produce active nitrogenase in plant mitochondria [114,115,116], and there is no doubt that in order to succeed, we need a well-founded and detailed understanding of the assembly, action, and regulation of this intriguing enzyme system. The story of nitrogenase is far from told.

## Data Availability

Not applicable.
